# Assessing the Role of Flexible Sigmoidoscopy for Patients With Positive Fecal Immunochemical Test (FIT) and Rectal Bleeding as a Sole Symptom Within the Fast-Track Pathway

**DOI:** 10.7759/cureus.69060

**Published:** 2024-09-10

**Authors:** Kareem Attia, Michael Lim, Dimitrios Pissas

**Affiliations:** 1 General and Colorectal Surgery, York and Scarborough Teaching Hospitals NHS Foundation Trust, Scarborough, GBR; 2 Colorectal Surgery, York and Scarborough Teaching Hospitals NHS Foundation Trust, York, GBR

**Keywords:** colorectal cancer, fecal immunochemical test, flexible sigmoidoscopy, rectal bleeding, significant bowel pathology, splenic flexure, two-week wait pathway

## Abstract

Background

Rectal bleeding is a frequent symptom, with causes ranging from benign conditions to serious diseases like colorectal cancer (CRC) and inflammatory bowel disease (IBD). In the UK, the two-week wait (2WW) referral pathway, which includes the fecal immunochemical test (FIT), plays a key role in triaging suspected CRC cases. This study evaluates the effectiveness of flexible sigmoidoscopy (FS) in detecting significant bowel pathologies (SBPs) in FIT-positive patients with isolated rectal bleeding.

Methods

We reviewed records of 344 patients with isolated rectal bleeding and a positive FIT result, referred to the 2WW pathway at York and Scarborough Teaching Hospitals NHS Foundation Trust from February to December 2023. All patients underwent colonoscopy. Findings from colonoscopy were used as a standard to compare with the expected reach of FS. Pathologies were categorized into SBPs (cancer, IBD, polyps ≥10 mm) and non-SBPs, and the location of SBPs in relation to the splenic flexure was also assessed.

Results

The average age of patients was 61.58 years. Significant bowel pathology (SBP) was identified in 89 of 344 (25.9%) patients, including 16 (18%) patients with cancer, 21 (23.6%) patients with IBD, and 52 (58.4%) patients with large polyps. All cases of cancer and IBD were found distal to the splenic flexure, while 21.1% (11/89) of large polyps were proximal. Higher FIT values (>100 µg Hb/g feces) and older age were significantly associated with SBPs. However, age and higher FIT values did not predict whether SBPs were proximal or distal.

Conclusion

For FIT-positive patients with isolated rectal bleeding, FS can serve as an effective initial diagnostic tool. Patients without detected cancer can be downgraded from the fast-track pathway to routine colonoscopy follow-up to avoid missing proximal premalignant lesions. This approach enhances resource utilization while ensuring comprehensive patient care. Further studies are needed to improve triage criteria and diagnostic accuracy within the 2WW pathway.

## Introduction

The presentation of a patient with rectal bleeding is prevalent in clinical practice and involves a reasonable proportion of the general population [1]. Epidemiological studies estimate that the prevalence of rectal bleeding ranges from 13% to 34% in community settings, yet a concerning observation is that less than half of individuals experiencing this symptom seek medical evaluation [2]. This underreporting highlights a potential gap in public awareness and underscores the importance of timely medical assessment for this symptom. Notably, significant causes of the condition vary, including benign conditions such as hemorrhoidal diseases and more severe pathologies, such as colorectal cancer (CRC) and inflammatory bowel disease (IBD). Therefore, these significant variations need a careful approach to diagnosis and its management to be effective in differentiating distinct etiologies [1].

The two-week wait (2WW) referral pathway for the investigation of suspected CRC has been standardized in the United Kingdom. This has expedited the way patients presenting with red flag symptoms are being evaluated, which in many cases have rectal bleeding [3,4]. The implementation of the fecal immunochemical test (FIT), a non-invasive test that detects a small amount of blood in fecal samples, in this evaluation, has helped to triage this population. The FIT possesses higher sensitivity for the evaluation of CRC risk as opposed to a history-based evaluation [5].

Despite this advance, there are still no firm recommendations on the best diagnostic approach for FIT-positive patients with isolated rectal bleeding and no other worrying features [6]. While FIT helps to identify patients at higher risk, it does not guide with the location of the lesion, which may lead to a lot of unnecessary colonoscopies. There is debate as to whether a colonoscopy is needed or whether the use of flexible sigmoidoscopy (FS) is robust enough to detect all potential causes of patients presenting with rectal bleeding. FS, limited by its design and bowel preparation, is an excellent tool for the detection of distal colorectal pathology, but its utility in this subgroup of patients remains uncertain. Indeed, prior to the availability of FIT, many patients with rectal bleeding would have had an FS only as the procedure of choice at the discretion of clinicians. Adding further complexity to clinical decision-making is the increasing incidence of CRC in younger patients with lower GI bleeding, which may or may not be on the left-hand side of the colon [6].

Our study examines the utility of FS for the detection of pathology in FIT-positive patients referred through the 2WW pathway who present solely with rectal bleeding as the sole symptom. We hope to help identify the additional potential criteria to assist in the process of triage under the current two-week referral framework. Through this piece of research, we intend to provide insight into how diagnostic accuracy and resources could be improved and utilized efficiently to maximize patient outcomes. 

## Materials and methods

Study population

The study population consisted of 344 patients with isolated rectal bleeding and a positive FIT result, consecutively referred by general practitioners to the 2WW pathway for the Colorectal Service at York and Scarborough Teaching Hospitals NHS Foundation Trust, United Kingdom, between February and December 2023. All patients in our study population underwent a full diagnostic colonoscopy.

To assess the utility of FS as a diagnostic tool in our patient population, we retrospectively analyzed colonoscopy findings as the reference standard. Colonoscopy allows for a comprehensive examination of the entire colon, including the proximal region beyond the reach of FS, providing a definitive diagnosis for all significant bowel pathologies (SBPs).

Exclusion criteria 

Patients with known IBD, polyposis syndromes, a previous diagnosis of CRC, colonic adenoma, acromegaly, a family history of familial adenomatous polyposis or hereditary non-polyposis CRC, or a strong family history of bowel cancer (indicated by two first-degree relatives with CRC or one first-degree relative with CRC when younger than 45 years of age), incomplete colonoscopies (failure to reach the caecum), and colonoscopies with inadequate bowel preparation were excluded to allow the analysis of patients who had an assessment of their whole colon.

Referral criteria and test protocol 

Patients with suspected CRC were referred via the 2WW pathway, a standardized referral system in the UK that aims to expedite the evaluation of individuals with "red flag" symptoms suggestive of cancer [3]. In this pathway, patients meeting specific criteria, such as rectal bleeding, are referred for urgent investigation within two weeks of presentation [3]. A positive FIT result, defined as ≥10 μg/g of fecal hemoglobin, was one of the criteria used for referral in our study. Patients referred through the 2WW pathway typically undergo colonoscopy or, in some cases, CT colonography. However, our study included only patients who had a colonoscopy.

Diagnostic procedures 

The colonoscopic findings were categorized into two groups: First, SBP included cancer, IBD, and polyps ≥ 10 mm, which were confirmed by histopathological assessment. Second, non-SBP included benign conditions not requiring urgent intervention. 

Furthermore, SBPs were categorized based on their location in relation to splenic flexure - proximal (beyond the splenic flexure and not within the reach of FS) or distal (within the reach of FS) to assess the proportion of SBPs potentially missed and detectable by FS if it was the sole diagnostic tool. 

Data collection and analysis 

Patient demographics data (age and gender), FIT value (quantitative value categorized as 10-100 or >100 µg of Hb/g of feces), and colonoscopy findings were collected from electronic hospital records. Although we did not stratify FIT values according to predefined clinical cut-off points, thus precluding relevant clinical information, this categorization also enabled us to assess whether FIT values within this range would correlate with other diagnostic categories. The descriptive statistics were expressed using means and standard deviations for continuous variables, while frequencies were used for categorical variables. 

Chi-square tests were employed to analyze the associations between categorical variables (age groups, FIT value groups, and lesion location) across the relevant comparisons. Independent t-tests were used to assess differences in mean age between the two groups, while one-way analysis of variance (ANOVA) tests were utilized to explore potential differences in the mean age across multiple groups. In cases where the ANOVA yielded a statistically significant result, Tukey's honestly significant difference (HSD) post-hoc test was employed to conduct pairwise comparisons between groups and identify which specific groups differed significantly from each other in terms of their means.

For statistical analysis, the IBM SPSS Statistics for Windows, Version 27.0 (IBM Corp., Armonk, NY) was used. Significance was set at a level of 0.05. 

Ethical considerations 

The study was registered as an audit with the Clinical Governance of Surgery Care Group at York and Scarborough Teaching NHS Foundation Trust (CG3/Surgery/PRbleed). Patient confidentiality was strictly maintained throughout the study, and data were anonymized and stored securely.

## Results

Demographic characteristics 

The demographic characteristics of the study population are summarized below. A total of 344 patients were included in the analysis. The mean age was 61.58 years (SD = 14.387), ranging from 20 to 91 years. This suggests that our study population is representative of the typical age range for CRC screening. The gender distribution was nearly equal, with 169 (49.1%) male and 175 (50.9%) female participants, strengthening the generalizability of our findings.

FIT value distribution 

The distribution of FIT values in our study population is as follows: 160 (46.5%) patients had FIT values between 10 and 100 µg Hb/g feces, while 184 (53.5%) patients had values exceeding 100 µg Hb/g feces. The high proportion of patients with FIT values exceeding 100 µg Hb/g feces underscores the potential need for further investigation in patients with high FIT values.

SBP

Eighty-nine (25.9%) patients in our study were diagnosed with SBP, while the remaining 255 patients (74.1%) did not have SBP. This relatively high prevalence of SBP emphasizes the importance of the triage strategies in the two-week pathway.

Type of SBP and its location in relation to the splenic flexure

Of the 89 patients with SBP, 16 (18.0%) patients had cancer, 21 (23.6%) patients had IBD, and 52 (58.4%) patients had large polyps. All cancer and IBD cases were located distal to the splenic flexure, while 11 of 52 patients with large polyps were located proximal to the splenic flexure. This distribution highlights the predominance of distal pathology in cancer and IBD cases, while polyps exhibit a more mixed distribution.

The findings from the preceding subsections are summarized in Table 1.

**Table 1:** FIT results for patients with rectal bleeding within the two-week wait pathway and corresponding findings of significant bowel pathology (SBP) in relation to the splenic flexure. SBP: significant bowel pathology, FIT: fecal immunochemical test, Hb: hemoglobin

Category	Subcategory	N	%
FIT value (µg Hb/g feces)	10-100	160	46.5
FIT value (µg Hb/g feces)	>100	184	53.5
SBP presence	Yes (SBP)	89	25.9
SBP presence	No (Non-SBP)	255	74.1
SBP type	Cancer	16	18
SBP type	IBD	21	23.6
SBP type	Polyps	52	58.4
SBP location	Proximal SBP	11	12.4
SBP location	Distal SBP	78	87.6

SBP vs. non-SBP: demographic characteristics and FIT value analysis 

Age Distribution

Patients with SBP were significantly older when compared to those without SBP (64.51 years, SD = 13.797 versus 60.55 years, SD = 14.475, p = 0.025). This suggests that age is a significant risk factor for SBP.

Gender Distribution and Analysis

The chi-square test revealed no significant association between gender and the presence of SBP (χ² = 0.314, df = 1, p = 0.575). The distribution of males and females was similar in both groups: within the SBP group, there were 46 male patients (51.7%) and 43 female patients (48.3%), and in the non-SBP group, there were 123 male patients (48.2%) and 132 female patients (51.8%).

FIT Value Analysis

**Figure 1:**
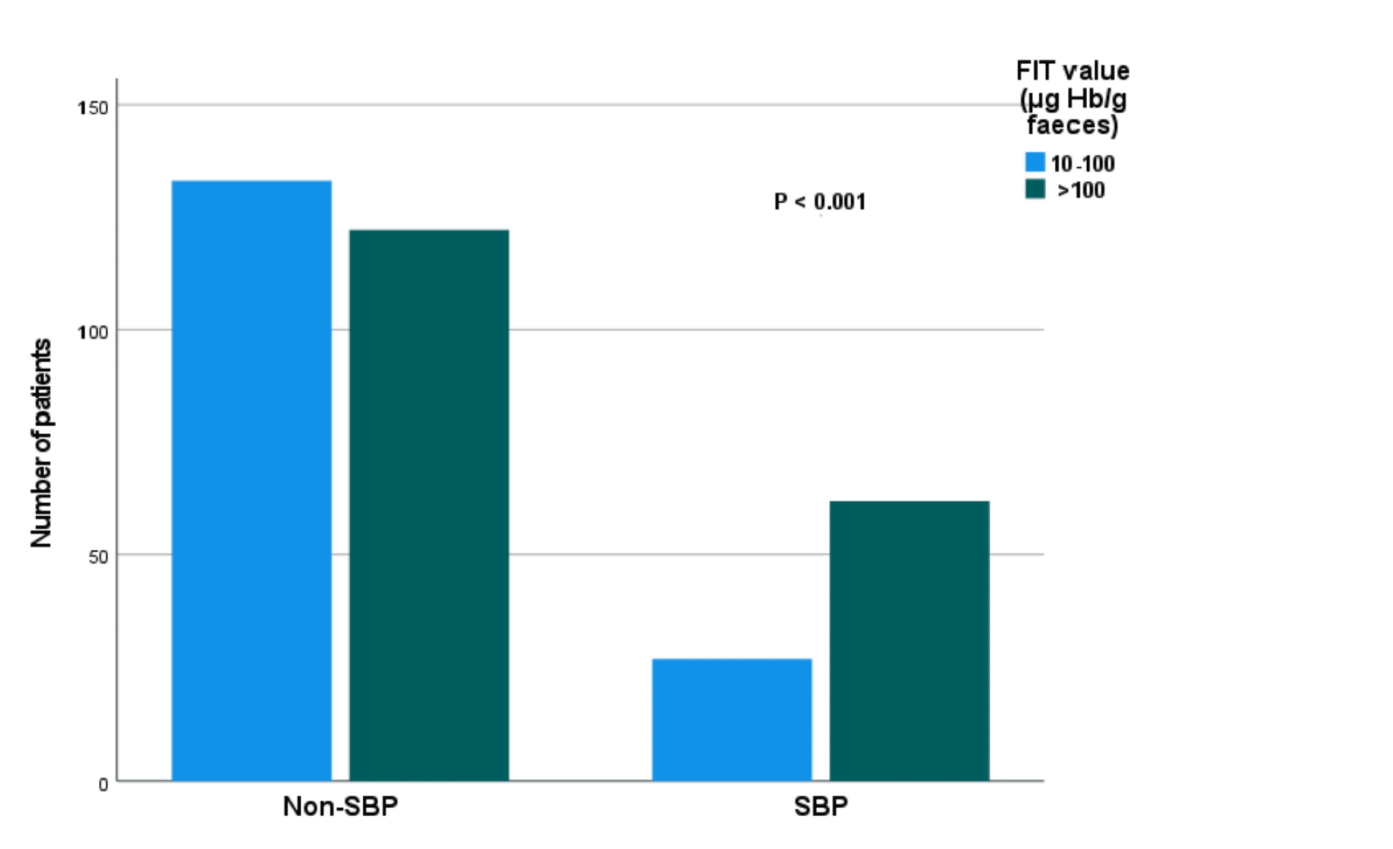
Distribution of values (μg Hb/g feces) in patients with and without significant bowel pathology (SBP). The difference in the fecal immunochemical test (FIT) value distribution between the two groups was assessed using a chi-squared test (p < 0.001).

**Figure 2:**
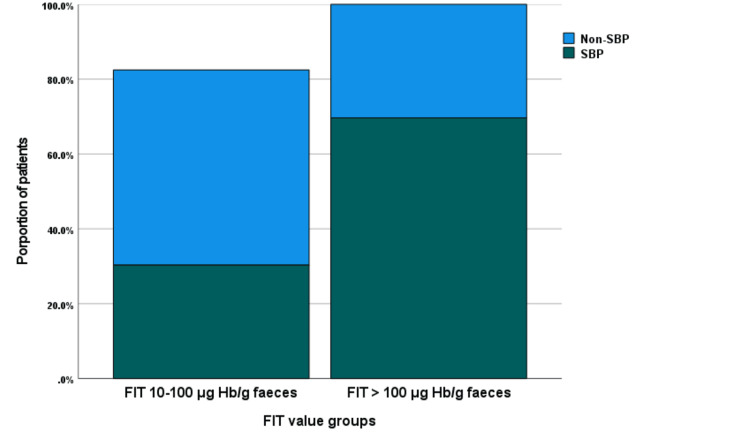
Proportion of patients with significant bowel pathology (SBP) by fecal immunochemical test (FIT) value groups

The distribution of FIT values among patients with and without SBP is shown in Figure 1. A greater proportion of patients with SBP (62, 69.7%) had FIT values >100 µg Hb/g feces, compared to 122 (47.8%) of patients without SBP. This difference was statistically significant (χ² = 12.626, df = 1, p < 0.001). This suggests the potential diagnostic utility of FIT in detecting SBP, as depicted in Figure 2.

Cancer vs. IBD vs. polyp: demographic characteristics, FIT value analysis, and location in relation to splenic dlexure 

Age Distribution

**Figure 3:**
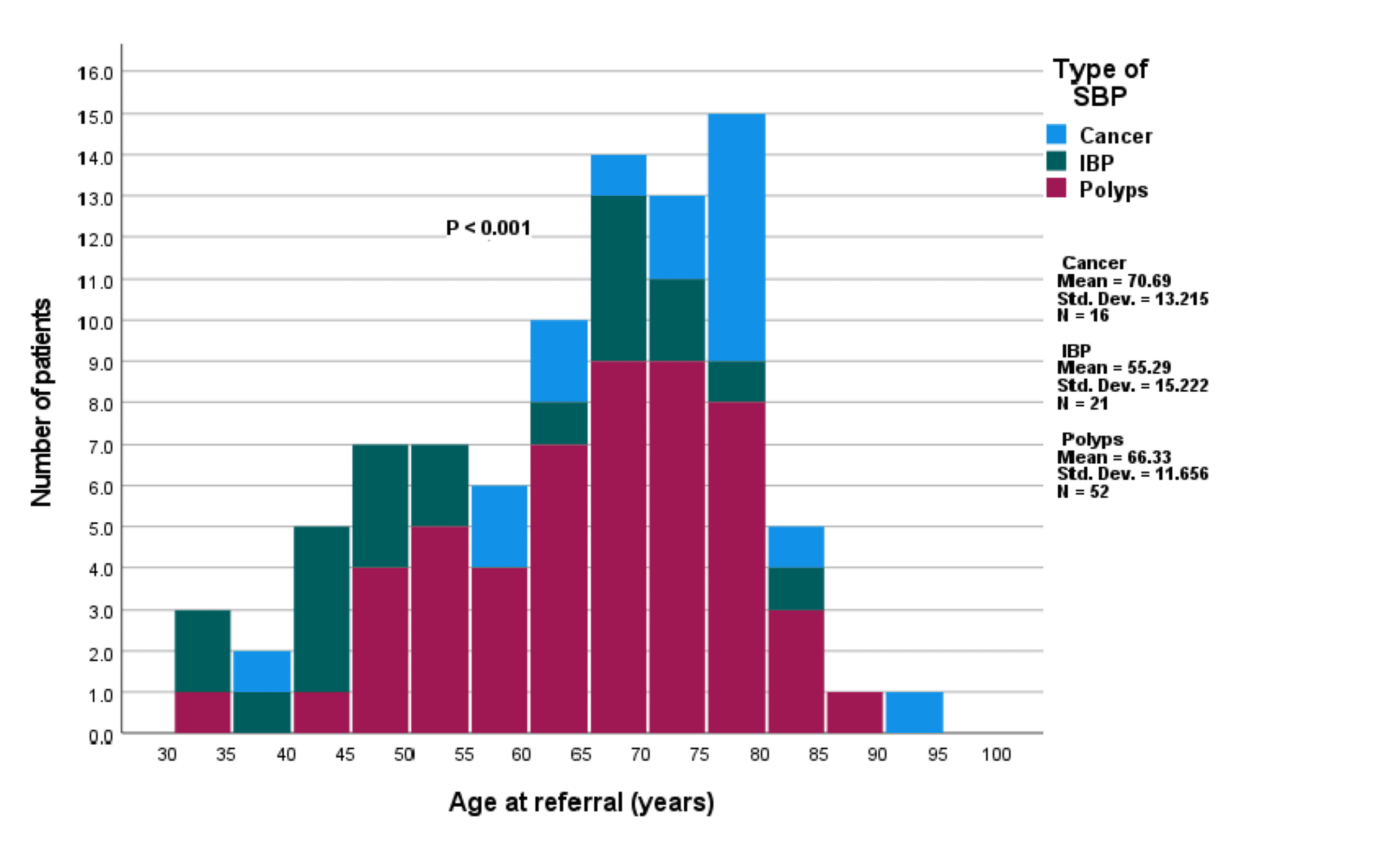
Age distribution by significant bowel pathology (SBP) subtype. The difference in mean age between the significant bowel pathology (SBP) subtypes was assessed using analysis of variance (ANOVA) (p < 0.001).

The analysis of variance (ANOVA) showed a significant difference in mean age at referral among patients with different SBP types: cancer (mean = 70.69 years, SD = 13.215), IBD (mean = 55.29 years, SD = 15.222), and polyps (mean = 66.33 years, SD = 11.656) (F = 7.789, p < 0.001) (Figure 3). Tukey HSD post-hoc tests confirmed that patients with cancer and polyps were significantly older than those with IBD (p < 0.001 and p = 0.004, respectively), while no significant age difference existed between the cancer and polyp groups (p = 0.464).

Gender Distribution and Analysis

The chi-square test indicated a marginally significant association between gender and SBP subtype (χ² = 5.994, df = 2, p = 0.050). This finding suggests a potential trend in SBP distribution based on gender: cancer (10/16, 62.5% male) and polyps (30/52, 57.7% male) may be slightly more prevalent in males, whereas IBD (6/21, 28.6% male) may be slightly more prevalent in females.

FIT Value Analysis

**Figure 4:**
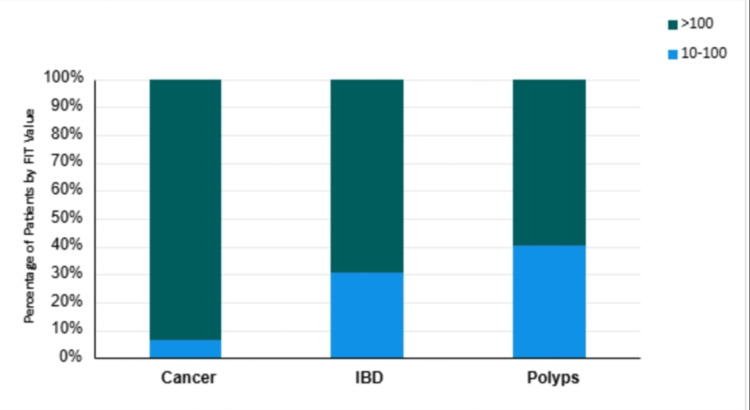
Distribution of FIT values (μg Hb/g feces) among SBP subtypes. The difference in the fecal immunochemical test (FIT) value distribution among the significant bowel pathology (SBP) subtypes (cancer, IBD, polyps) was assessed using a chi-squared test (p = 0.026).

The distribution of FIT values among patients with different types of SBP is shown in Figure 4. Most patients with cancer (15/16, 93.8%) had FIT values >100 µg Hb/g feces, compared to 16/21 (76.2%) of patients with IBD and 31/52 (59.6%) of patients with polyps. This difference was statistically significant (χ² = 7.300, df = 2, p = .026). This finding underscores the potential value of FIT as a diagnostic tool, particularly in differentiating between SBP subtypes and identifying individuals who might be at higher risk for CRC. 

Location in Relation to the Splenic Flexure Analysis

**Figure 5:**
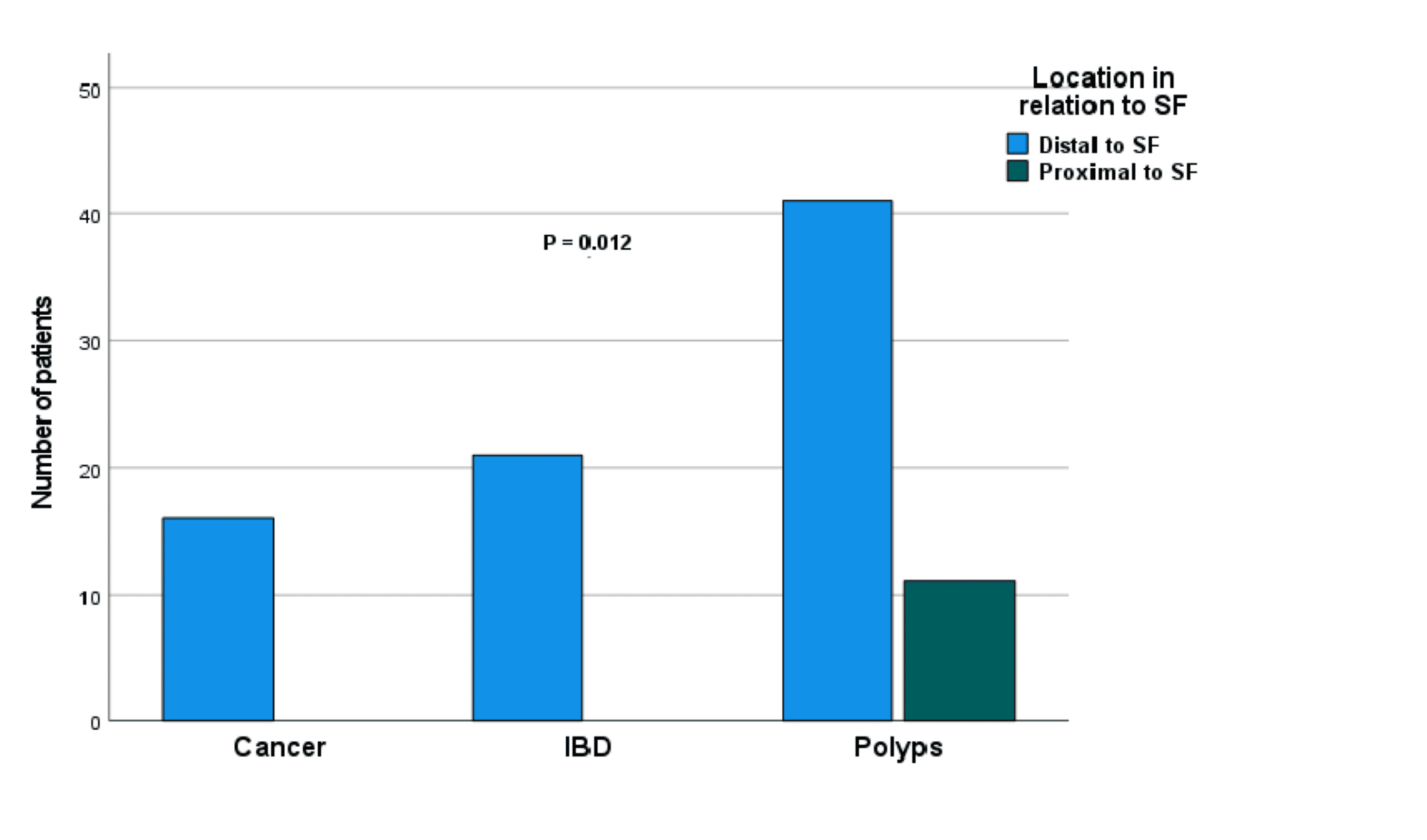
Distribution of location in relation to splenic flexure (SF) among the SBP subtypes. The association between significant bowel pathology (SBP) subtypes and their location was assessed using chi-squared tests (p = 0.012).

The chi-square tests revealed a significant association between SBP subtype and lesion location relative to the splenic flexure (χ² = 8.931, df = 2, p = 0.012) (Figure 5). Notably, all cases of cancer (100%) and IBD (100%) were identified distally. By contrast, polyps exhibited a distribution across both regions, with 41 cases (78.8%) located distally and 11 cases (21.2%) proximally. This finding demonstrates the distinct distribution patterns of different SBP types, with IBD and cancers concentrated distally. 

Proximal SBP vs. the combined group of distal SBP and non-SBP: demographic characteristics and FIT value analysis 

Age and Age Group Distribution and Analysis

Patients with proximal SBP were significantly older on average (mean = 71.18 years, SD = 8.784, n = 11) compared to those in the combined distal SBP and non-SBP group (mean = 61.26 years, SD = 14.435, n = 273), as confirmed by the t-test (t = -2.264, df = 342, p = 0.024).

Gender Analysis 

The chi-square test revealed no significant association between gender distribution and pathology type (proximal SBP vs. combined distal SBP and non-SBP) (χ² = 0.133, df = 1, p = 0.715). The distribution of males and females was similar between patients with proximal SBP (six males (54.5%), five females (45.5%)) and those in the combined group (163 males (49.2%), 170 females (50.8%)). 

FIT Value Analysis

For patients with proximal SBP, five cases (45.5%) had an FIT value of 10-100, and six cases (54.5%) had an FIT value of more than 100. In the combined group of distal SBP and non-SBP, 155 cases (46.6%) had an FIT value of 10-100, and 178 cases (53.4%) had an FIT value of more than 100. 

The chi-square test results showed no significant association between FIT value and the type of pathology (proximal SBP vs. distal SBP and non-SBP) (χ² = 0.005, df = 1, p = 0.943). This suggests that there is no significant difference in the FIT value distribution between patients with proximal SBP and those with distal SBP and non-SBP. This finding highlights the need for further exploration of diagnostic strategies or risk factors to support accurate triage. 

## Discussion

Our study investigated patients who were referred within the suspected CRC pathway and examined the interaction between age, FIT value, and colonoscopic findings of significant bowel pathology in relation to the splenic flexure. There was a significant pattern noticed regarding the age as patients with SBP were older on average; that pattern included also proximal SBP in comparison to the combined distal/non-SBP group. Moreover, patients with cancer or polyps were older than those with IBD. These observations align with the established understanding that the risk of CRC, a major component of SBP, escalates with age, with over 40% of new cases in the UK occurring in individuals aged 75 and over [7].

While our study showed that patients with SBP were older on average, suggesting age as a potential risk factor for SBP, the absence of a specific age cut-off that reliably differentiates between those with and without SBP in our study population highlights the need to consider additional factors beyond age for risk assessment. This observation is consistent with the findings of Johnstone et al. [8], who also emphasized the need to consider multiple factors when evaluating patients with rectal bleeding. Furthermore, Johnson et al. (2013) found that while age was associated with a higher risk of raised FIT value in patients with significant bowel disease, it was not a strong predictor in those without pathology, suggesting that age alone might not be sufficient for accurate risk stratification. These findings, along with the observations of Digby et al. (2019) regarding the limitations of the fecal hemoglobin, age, and sex test (FAST score), underscore the importance of incorporating other clinical and diagnostic information, such as FIT values and patient symptoms, to improve the accuracy of triage in the 2WW pathway [9]. 

Higher FIT values were also seen in patients with SBP as a greater proportion of patients in this group had FIT values exceeding 100 ug Hb/g feces. This finding is consistent with the work of Farrugia et al. [10], who demonstrated the superior accuracy of FIT in comparison to symptom-based referral criteria, supporting the diagnostic value of FIT in identifying patients who may benefit from further investigation. Higher FIT values were more frequent in patients with cancer than polyps and IBD, which could potentially help in the identification of patients with a higher risk of CRC. Our findings are in keeping with that of Hicks et al. [11], who demonstrated high sensitivity of FIT for detecting CRC in patients presenting with rectal bleeding. However, the proportion of patients with high FIT value was approximately similar in proximal SBP and combined distal/non-SBP, which could be due to the high FIT value of cancer located distally. While age and FIT value can help in identifying high-risk patients, they could not be reliably used to identify the location of the lesion in relation to splenic flexure. 

The key finding was the distribution of SBP subtypes as all cancers, IBD, and the majority of polyps were detected distally while only 21.1% of large polyps were located proximally. This is in keeping with the reports by Cross et al. in relation to the predominance of distal pathology in patients who present with isolated rectal bleeding [12]. This emphasizes the use of FS in this cohort to identify CRC. Although a small proportion of precancerous lesions (polyps) can be missed if we rely on FS only, it could be used as an initial urgent investigation within the two-week pathway, which can be followed by routine colonoscopy to avoid missing proximal polyps. 

There are several limitations in our study. First, this is a retrospective single-center study, and our findings may not be generalized to another population and healthcare setting. Moreover, with a small sample size of 344 patients, there may be weaknesses in our analyses that do not allow us to detect subtler relationships between variables. Furthermore, different thresholds and cut-off values for FIT and age may allow a more granular analysis to obtain a better understanding of interactions between factors. We also did not study FIT-negative patients during the same time interval, this could have provided insights into the prevalence of SBPs or non-SBP findings in this population. In addition, our study assumed the outcomes of an ideal FS based on successful colonoscopy results. This means that we presumed FS would reach up to the splenic flexure, which is not always the case in practice. We did not perform FS followed by colonoscopy within our cohort, so our findings are based on theoretical assumptions rather than direct observations. This assumption introduces potential inaccuracies in our conclusions regarding FS effectiveness and the detection rates of significant pathologies. Further larger prospective multicentric studies with large patient numbers and diverse populations are needed to provide robust data. 

## Conclusions

In summary, this study has highlighted that FS can be a useful initial diagnostic tool in patients presenting with isolated rectal bleeding and positive FIT results within the two-week pathway. The likelihood of cancer being missed is low but one in five patients will have large polyps in the right colon beyond the reach of a high-quality FS. In those patients with abnormal FIT measurements, a normal FS may warrant a completion colonoscopy, but this could be done with the fast-track pathway allowing resource allocation and planning of services at a time when capacity for urgent colonoscopy is at a premium. Future research will be directed to defining the added clinical or biomarker-based criteria that best serve to enhance the identification of proximal SBPs and further refine diagnostic strategies based on individual patient risk profiles. This should ultimately enhance the detection of lesions, patient outcomes, and resource optimization within the urgent referral pathway.
